# Bioactivity Potential of *Aesculus hippocastanum* L. Flower: Phytochemical Profile, Antiradical Capacity and Protective Effects on Human Plasma Components under Oxidative/Nitrative Stress In Vitro

**DOI:** 10.3390/ph14121301

**Published:** 2021-12-14

**Authors:** Aleksandra Owczarek, Joanna Kołodziejczyk-Czepas, Paulina Marczuk, Julia Siwek, Katarzyna Wąsowicz, Monika Anna Olszewska

**Affiliations:** 1 Department of Pharmacognosy, Faculty of Pharmacy, Medical University of Lodz, 90-151 Lodz, Poland; julia.siwek@stud.umed.lodz.pl (J.S.); katarzyna.wasowicz6@gmail.com (K.W.); monika.olszewska@umed.lodz.pl (M.A.O.); 2 Department of General Biochemistry, Faculty of Biology and Environmental Protection, University of Lodz, 90-236 Lodz, Poland; joanna.kolodziejczyk@biol.uni.lodz.pl (J.K.-C.); mmarczuk.paulina@gmail.com (P.M.)

**Keywords:** *Aesculus hippocastanum*, flavonoids, astragalin, kaempferol glycosides, human plasma

## Abstract

Horse chestnut (*Aesculus hippocastanum*) flower is a traditional medicine applied to alleviate symptoms of chronic venous insufficiency (CVI). However, its flavonoid-based composition has not been sufficiently recognized, and the data supporting its traditional application are lacking. In the work, 43 constituents were detected by UHPLC–PDA–ESI–TQ–MS/MS (flavonoids, phenolic acids, flavanols, and coumarins), including 31 reported in the flower for the first time. The quantitative HPLC–PDA study (developed and validated for quality control purposes) indicated the fractionated extraction as an efficient method for enhancing the total polyphenol content (TPHC) in the extracts (up to 414.06 mg/g) and kaempferol glycosides as their dominant constituents (75.05–82.14% TPHC). The activity studies showed significant scavenging properties of the extracts and their constituents towards reactive oxygen species (especially against highly reactive hydroxyl radical, with capacities up to 7.85 mmol ascorbic acid equivalents/g). Moreover, the analytes relevantly protected human plasma biomolecules from peroxynitrite-induced oxidative/nitrative damage; at 1–50 µg/mL, they hindered the protein nitration and lipid peroxidation, decreasing the levels of 3-nitrotyrosine (by up to 50%) and thiobarbituric acid reactive substances (by up to 70%), respectively. The extracts also averted the depletion of plasma thiols (by up to 67%) and improved the non-enzymatic antioxidant capacity of plasma. The demonstrated mechanisms might be partly responsible for the efficacy of the flower in CVI. Additionally, the anti-aggregatory and anticoagulant properties of the extracts were found only mild or negligible, which suggests that they may be safely applied with drugs impacting the coagulation process.

## 1. Introduction

Horse chestnut (*Aesculus hippocastanum* L.) is a large deciduous tree, originating from small areas of the Balkan Peninsula but nowadays spread widely throughout the temperate zone of the Northern Hemisphere [1]. It owes its popularity mainly to its ornamental value and medicinal properties. Among plant tissues applied in official and traditional medicine are the seed, bark, and flower of horse chestnut. All the plant materials are prescribed chiefly in chronic venous disorders, lower leg varicose veins, and hemorrhoids; they differ, however, in terms of composition and potential mechanism of action [2]. Undoubtedly, the most recognized and studied is the seed, containing a mixture of triterpenoid saponins, escin, which is responsible for the seed’s clinical effects, mainly due to potent anti-inflammatory activity [3]. On the other hand, the data supporting the use of bark and flower are less extensive, and there are still information gaps to be filled.

In the previous paper, we studied more closely the rationale behind the efficacy of the extracts from horse chestnut bark, which contains high amounts of coumarins and flavanol derivatives [4]. The effectiveness of the flower is also believed to be phenolic-based, and the thus-far published studies indicate a flavonol-oriented profile. To date, seven derivatives of kaempferol and quercetin have been isolated from the plant material, namely, kaempferol 3-*O*-arabinofuranoside, kaempferol 3-*O*-glucopyranoside, kaempferol 3-*O*-rhamnopyranoside, kaempferol 3-rutinoside, quercetin, quercetin 3-*O*-glucopyranoside, and quercetin 3-rutinoside [5]. The only reported HPLC study allowed for identification of up to 10 constituents (including six previously isolated); however, the reported chromatogram indicates the presence of a larger number of compounds, most of which remained unidentified [6].

Varicose veins and related complaints, which are the main indication for the horse chestnut application, have a complex and not yet fully explained etiology. Nevertheless, oxidative stress is listed as one of the possible causes or exacerbating factors [7]. A growing body of evidence demonstrates increased levels of reactive oxygen species (ROS), e.g., O_2_^•−^, depletion of the endogenous antioxidative system, and intensified oxidative modifications of proteins and lipids in the varicose veins itself [7,8], as well as in the blood of patients with this condition [9]. The disturbances, marked by the changes in the levels of particular oxidative stress markers (e.g., thiobarbituric acid reactive substances, TBARS, a marker of lipid peroxidation) are connected with increased risk of vascular complications [10,11]. The antioxidant activity, including direct ROS scavenging and protecting biomolecules from oxidative and nitrative damage, might thus be one of the beneficial effects of plant remedies used in prophylaxis and treatment of venous disorders. Such activity is often connected with phenolics, including flavonoids [12]; thus, it might be also one of the mechanisms of horse chestnut flower efficacy.

Blood stasis and vessel damage, often accompanying venous insufficiency, might cause thrombotic complications such as superficial venous thrombosis or deep vein thrombosis in more severe cases [13]. Therefore, anti-aggregatory and anti-coagulant properties of natural compounds might also play a role in alleviating symptoms or decreasing the risk of complications [12]. On the other hand, such properties might cause unexpected interactions with already taken medications, such as in the case of extracts from *Ginkgo biloba* or garlic [14]. Therefore, the evaluation of the plant material capacity in those directions is important for the assessment of its safety. 

Considering the abovementioned premises, the aim of the study was a thorough investigation of the composition of horse chestnut flower in terms of potentially active phenolics and an initial evaluation of some of the activity mechanisms that might be relevant for its application in venous disorders. The studies were performed on the methanolic extract from the flower and its fractions with enhanced phenolic content obtained by fractionated extraction. The detailed qualitative and quantitative studies were performed by UHPLC–PDA–ESI–TQ–MS/MS and HPLC–PDA methods, respectively. The antioxidant capacity tests included scavenging of the most ubiquitous ROS present in human organism that might play a role in developing vascular oxidative stress. Furthermore, protective effects of the extracts towards proteins and lipids of human plasma and their impact on non-enzymatic antioxidant capacity of plasma (NEAC) were studied in a biological model reflecting oxidative/nitrative stress conditions. The basic anti-aggregatory and anticoagulant tests comprised anti-platelet effects in platelets activated by collagen and ADP as well as influence on blood clotting times. 

## 2. Results and Discussion

In the first part of the study, crude methanolic extract (ME) from the horse chestnut flower was obtained. Due to the universal extraction properties of methanol towards phenolics, the composition of ME reflect the native phenolic composition of the flower. Then, to further concentrate the active constituents, we performed fractionated extraction of ME, which yielded organic fractions of different polarities, i.e., diethyl ether fraction (DEF), ethyl acetate fraction (EAF), and *n*-butanol fraction (BF). The specific polarities of the solvents enable selective concentration of constituents, enhancing the phenolic content and facilitating the identification by giving prominence to the minor components of the crude extract.

### 2.1. Qualitative UHPLC–PDA–ESI–TQ–MS/MS Profiling

**Figure 1:**
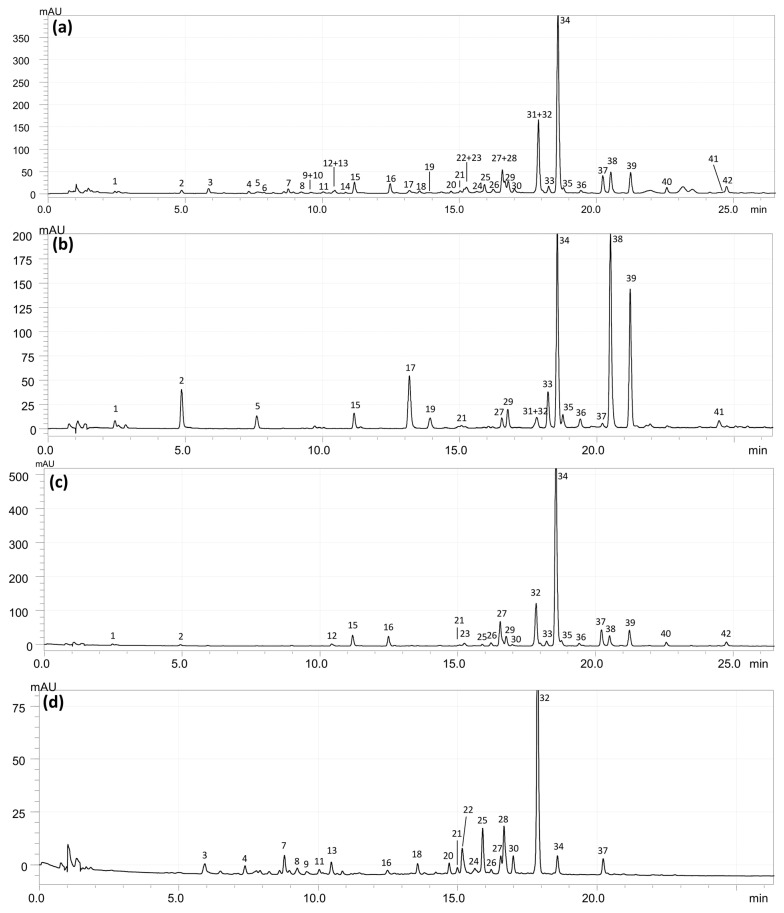
Representative UHPLC chromatograms of the investigated dry extracts from *A. hippocastanum* flower, λ = 280 nm. (**a**) ME, methanol extract; (**b**) DEF, diethyl ether fraction; (**c**) EAF, ethyl acetate fraction; (**d**) BF, *n*-butanol fraction. Peak numbering according to Table 1.

The qualitative phytochemical analysis of the investigated extracts was performed using the UHPLC–PDA–ESI–TQ–MS/MS method. A total of 43 different compounds were detected, and 42 of them were tentatively or fully identified by comparison of their chromatographic (retention time) and spectral information (UV–VIS, MS/MS) with that of authentic standards and literature data (Table 1, Figure 1). The identified compounds belonged to several chemical classes, including simple phenolic acids, their derivatives (pseudodepsides and spermidine conjugates), coumarins, mono- to trimeric flavanols, and flavonol glycosides and their aglycones. A total of 31 of the detected compounds were found for the first time in the *A. hippocastanum* flower, and 19 in the species in general (Table 1).

Among simple phenolic acids, gallic acid (**1**), protocatechuic acid (**2**), *p*-hydroxybenzoic acid (**5**), caffeic acid (**10**), and *p*-coumaric acid (**17**) were identified by comparison with authentic standards. Peak **19** with [M−H]^−^ ion at *m/z* 163, identical to that of *p*-coumaric acid, was tentatively identified as a coumaric acid isomer. As the comparison with the authentic standard of two other *trans*-isomers, i.e., *m*-coumaric acid and *o*-coumaric acid, did not show a match, this would probably be *cis*-p-coumaric acid. Additionally, four compounds with [M−H]^−^ ion at *m/z* 487 (**4**, **7**, **9**, and **13**) were found, and due to the presence of product ions at *m/z* 163 [coumaric acid −H]^−^ and 145 [coumaric acid − H_2_O − H]^−^, they were tentatively identified as derivatives of coumaric acid. Other derivatives of phenolic acids included caffeic acid pseudodepsides—chlorogenic (**3**) and cryptochlorogenic (**8**) acids that were identified by comparison with authentic standards. Moreover, conjugates of caffeic and ferulic acid with spermidine were detected. On the basis of the *m/z* value of their deprotonated molecule and consecutive neutral losses of −162 amu (caffeoyl moiety) or −176 amu (feruloyl moiety), we identified them as tricaffeoyl-spermidine (**40**) and dicaffeoyl-feruloyl-spermidine (**42**).

Phenolic acids are common constituents of plants and usually accompany flavonoids as their biogenetic precursors [15]. Specifically, spermidines are often found in flowers of different species and are believed to mostly come from pollen [16]. However, apart from caffeic acid glucoside [6], phenolic acid derivatives have not been reported in the *A. hippocastanum* flower to date. On the other hand, a couple of compounds from this group have been detected in the leaves of horse chestnut [17]. Nevertheless, this is the first report on the occurrence of all the above-described constituents in the plant.

Six of the detected compounds (**12**, **15**, **16**, **21**, **23**, and **29**) had a maximum in UV spectra at about 278 nm, characteristic of flavanols. Among them, on the basis of the *m/z* values of the [M−H]^−^ ions, we distinguished one monomer (**15**), four dimers (**12**, **21**, **23**, and **29**), and one trimer (**16**). Peaks **15** and **29** were identified with authentic standards as (−)-epicatechin and procyanidin A2, respectively, previously found in the bark of horse chestnut [18]. Additionally, peak **12** was confirmed with authentic standard as procyanidin B2, reported previously from the leaves [17]. Apart from the bark, several proanthocyanidins have been isolated from *A. hippocastanum* fruit shells [19,20], but this work is the first report on this group of compounds in the flower. 

**Figure 2:**
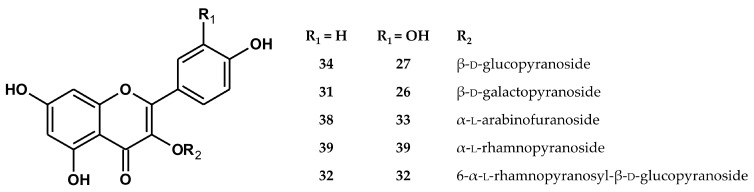
Structures of the main flavonoid glycosides identified in the extracts from the flowers of *A. hippocastanum*.

Flavonoids of the flavonol type (**20**, **22**, **24**–**39**, **41**, and **43**) were the most numerous constituents of the extracts (Figure 2). They included glycosides of kaempferol and quercetin that were discriminated on the basis of their product ions coming from the aglycon at *m/z* 285/284 and 301/300, respectively. The presence of free aglycones kaempferol (**43**) and quercetin (**41**) was also confirmed with authentic standards. Most of the monoglycosides were identified with the authentic standards, except for **36** and **37**, which were only partly identified as kaempferol pentoside and kaempferol acetylhexoside, respectively. Among the diglycosides, 3-rutinosides of kaempferol (**32**) and quercetin (**25**), as well as quercetin 3-sophoroside (**18**), were confirmed with authentic standards. Four further diglycosides (**20**, **24**, **28**, and **20**) were assigned as pentoside-hexosides and hexoside-rhamnosides of kaempferol and quercetin, judging by the neutral losses of −132 amu (pentose moiety), −162 amu (hexose moiety), or −146 amu (rhamnose moiety). The detected hexoside-rhamnosides were compared with authentic standards of 3-*O*-(4″-*O*-β-d-glucopyranosyl)-α-l-rhamnopyranosides of kaempferol and quercetin, previously found in the seed of horse chestnut [21,22]; however, in neither case did the retention times overlap. 

The present work confirmed the occurrence of seven flavonoids previously isolated from the plant material and two whose presence was chromatographically inferred [5]. However, it led also to the detection of 11 others that have not been reported from the flower. Several of this compounds have also been present in the leaves of horse-chestnut [17]. 

Among coumarins, only one constituent (**11**) was detected, and its identity was confirmed as fraxin by comparison with an authentic standard. Fraxin is a known component of *A. hippocastanum* bark, and its aglycon fraxetin have been isolated previously from the flower together with other coumarin aglycones, i.e., esculetin and scopoletin [23]. In the present work, the aglycones were, however, not detected. 

### 2.2. Quantitative HPLC–PDA Analysis 

The qualitative analysis of polyphenols in the extracts was carried out by the HPLC–PDA method developed and optimized to obtain satisfactory resolution of the investigated peaks. Due to the close structural similarity between many of the extracts’ constituents, the separation was carried out using fused-core HPLC column and ternary solvent system (composed of orthophosphoric acid, acetonitrile, and tetrahydrofuran). The validation of the method demonstrated its satisfactory linearity, sensitivity, precision, and accuracy (for details of method optimization and validation, see Supplementary Materials Tables S1–S3).

The obtained results (Table 2) constitute the first detailed quantitative profile of the individual polyphenols of horse chestnut flower. In the previous paper, the phenolics and flavonoids were determined using spectrophotometric methods only [6]. The total phenolic content in hydromethanolic extract from the flower obtained in that study by the Folin–Ciocalteu method was 88.24 mg of chlorogenic acid equivalents/g and was similar to the HPLC-determined value from our study that for ME was 74.26 mg/g. On the other hand, the content of flavonoids determined previously was only 15 mg of quercetin equivalents/g, while in our study, it reached 65.58 mg/g. Apart from the normal environmental variability of the raw plant materials, this discrepancy might be caused by differences in the specificity of the methods.

In comparison to the methanolic extract from *A. hippocastanum* bark (containing over 400 mg/g of phenolics) [4], ME from the flower was found to be less rich in polyphenolic compounds. However, further concentration of the flower phenolics might be achieved by enhancing the extraction procedure. One of the methods is liquid–liquid fractionation that allows for obtaining fractions enriched in phenolics of specific polarity. In our previous works, this method has been proven to be effective in selective extraction of polyphenols from *Prunus spinosa* flower [24], among others. Moreover, in the present work, the content of polyphenols was largely increased by fractionation; up to 310.56 mg/g and 414.06 mg/g in DEF and EAF, respectively. Such content is comparable with that obtained for the bark, but also, for example, with commercial grape seed extract, which has been previously shown to possess potent anti-oxidant and anticoagulant properties [25,26].

Due to the use of solvents of different polarities, the fractionation of the extract led also to some changes in relative proportions between particular constituents and groups of constituents (Table 2). Almost 80% of the methanolic extract investigated in the present study constituted kaempferol derivatives, with a small share of derivatives of quercetin (about 10%), flavanols (about 10%), and phenolic acids (about 2%). Two main constituents were kaempferol 3-glucoside (29.38 mg/g) and 3-rutinoside (16.31 mg/g), accounting for over 55% of the extract. The ratio between mono- and diglycosides, in general, was 2:1. In the obtained fractions, kaempferol derivatives were still prevailing; however, the DEF contained an increased proportion of simple phenolic acids (almost 10% of all phenolics) and EAF of flavanols (about 14%). The most visible difference between the extracts was the ratio between flavonoid mono- and diglycosides. Monoglycosides prevailed in DEF and EAF, and their proportion was higher than that in ME, i.e., 42:1 and 5:1, respectively. On the other hand, BF contained mainly diglycosides, with mono- to diglycosides ratio of about 1:8 and kaempferol 3-rutinoside as the dominant constituent (81.97 mg/g, 56% of the fraction).

When the possible variations in the activity and bioavailability of particular constituents are considered, the differences in the composition of the fractions might be relevant for their effects in vivo. For example, simple phenolic acids, such as those present in DEF, are usually easily absorbed [15]. On the other hand, the absorption of flavonoid glycosides depends strongly on the sugar moiety, and, for example, it is much faster and higher for glucosides than rutinosides [27]. In that context, the fractionated extraction using diethyl ether and ethyl acetate allows for concentrating the phenolics of horse chestnut flower and improves their proportions in terms of bioavailability.

### 2.3. Antioxidant Activity in Chemical Models

Oxidative stress is considered one of the causes of venous disorders, being the underlying factor of the complaints such as varicose veins or hemorrhoids [28], the main indications of the horse chestnut flower. Thus, the antioxidant activity of the flower constituents may be one of the mechanisms of action behind their efficacy. Previous studies have shown thus far that *A. hippocastanum* flower extract decreases the ROS generation in endothelial cells in vitro [6]. ROS are signaling molecules, vital for the proliferation and survival of the cells, but when produced in an excessed amount, they may damage the essential cellular components, such as proteins, lipids, and deoxynucleic acids [29]. The resulting structural changes impair the physiologic function of the biomolecules and may start or exacerbate the pathological processes, leading to the development or progress of vascular complaints [7]. One of the mechanisms by which the extracts’ constituents may diminish the cellular ROS levels is their direct scavenging.

**Figure 3:**
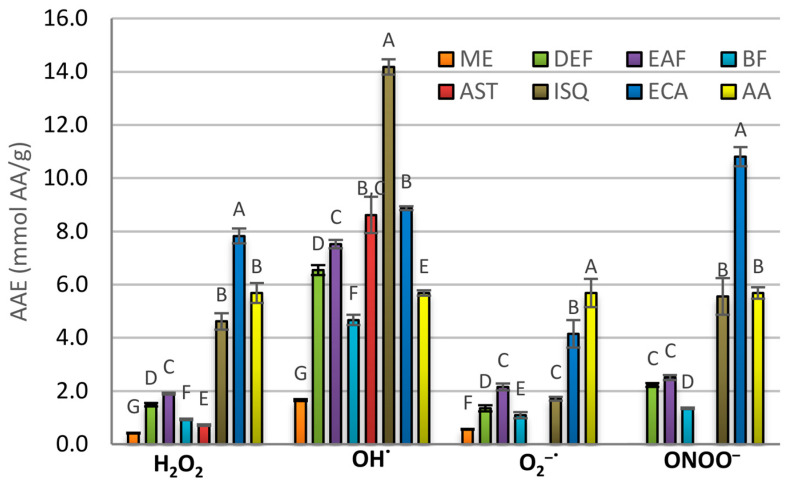
Scavenging activity of the extracts from the flower of *A. hippocastanum* and its selected constituents towards reactive oxygen species (ROS), expressed as ascorbic acid equivalents (AAE). Results presented as means ± SE (*n* = 5). ANOVA and post hoc tests for multiple comparisons were run foreach ROS separately, and the values statistically different at α = 0.05 were labeled with different capital (A–G). Analytes: ME—methanol extract; DEF—diethyl ether fraction; EAF—ethyl acetate fraction; BF—*n*-butanol fraction; AST—astragalin; ISQ—isoquercitrin; ECA—(−)-epicatechin; AA—ascorbic acid.

In the present study, we investigated the quenching potential of the extracts and their selected constituents towards some of the most relevant ROS operating in vivo, including hydroxyl radical (OH^•^), superoxide radical (O_2_^•−^), hydrogen peroxide (H_2_O_2_), and peroxynitrite (ONOO^–^). The results showed that all analytes scavenged the investigated ROS in a concentration-dependent manner (Table 3, Figure 3). The highest capacity in all tests was exhibited by the fractions richest in polyphenols, i.e., EAF and DEF. Depending on the ROS, those fractions presented the EC_50_ values of 15.22–154.02 µg/mL and 24.18–173.42 µg/mL, respectively, constituting about 25–130% of the activity presented by ascorbic acid (AA). As the main non-enzymatic plasma antioxidant, AA might serve as a valuable reference point for evaluating the potential exogenous antioxidants. Considering the results expressed in millimolar AA equivalents (AAE, mmol AA/g), the most potent capacity of the extracts and their constituents was towards OH^•^ radical (Figure 3). The capacity of DEF and EAF in that assay reached 6.54 mmol AA/g and 7.52 mmol AA/g compared to 5.82 mmol AA/g obtained for AA itself. Moreover, all investigated pure compounds had in that assay activity higher than that of AA.

Interestingly, the investigations on the selected pure polyphenols indicated that most of the extracts’ efficacy might come from the less abundant constituents, i.e., quercetin and flavanol derivatives. Specifically, in all assays, isoquercitrin and (−)-epicatechin were significantly more active than astragalin (Table 3, Figure 3). This fact is connected with the lack of ortho-dihydroxy structure in the molecule of kaempferol, responsible for improved delocalization of unpaired electrons and better stabilization of a flavonoid radical [30]. In the context of potential quality control, this observation might indicate the need for a specific quantitative assessment of quercetin derivatives. However, further analyses are also required to evaluate the behavior of particular constituents under more complex conditions, in which additional interactions with cellular components may impact their relative activity strength.

The ROS investigated in the present work are some of the most ubiquitous in vivo-operating species and might impact the development of varicose veins on several different levels. For example, O_2_^•−^ is the main factor causing NO depletion, one of the complications connected with endothelial dysfunction [31]. Moreover, in a rapid reaction between O_2_^•−^ and NO, a strong nitrative/oxidative agent arises, i.e., ONOO^–^ [7]. ONOO^–^ is a very destructive species that, among other effects, causes thiol degradation and nitration of aromatic compounds including amino acids [32]. Similar potent reactivity distinguishes OH^•^, towards which the analytes presented the highest quenching potential relative to AA. In the living organism, OH^•^ is formed in the Fenton reaction between H_2_O_2_ and ferrous ions. As in the case of ONOO^–^, harmful effects of OH^•^ may include enzyme inhibition and lipid peroxidation [32]. Direct damage of cell lipid membranes caused by ROS leads to endothelial injury and may cause vascular or valve dysfunction [7]. The research on varicose veins patients revealed increased levels of O_2_^•−^ in disabled veins [7]. Moreover, the elevated iron levels present in the insufficient veins may predispose the patients to increased production of OH^•^ [33]. Thus, the scavenging properties of the horse chestnut flower extract and its constituents may lessen the burden of ROS overproduction in such patients and diminish its harmful effects. To study more closely this hypothesis, in the next step of our study, we investigated the effects of the extract in a biological system of human plasma.

### 2.4. Antioxidant Activity in Plasma Model

The harmful influence of ROS may be traced in biological systems by assessing the levels of specific parameters. In the plasma of patients with varicose veins and prepared insufficient vein fragment, several such indicators of oxidative stress have been found. The evidence suggested, among others, an escalation of lipid peroxidation, which was evidenced by increased levels of malondialdehyde [9,10,11,28]. Other researchers also observed thiol depletion [9,34], decreased values of NEAC, and increased protein nitration, as indicated by the elevated levels of 3-nitrotyrosine (3-NT) [8,9,35,36].

**Figure 4:**
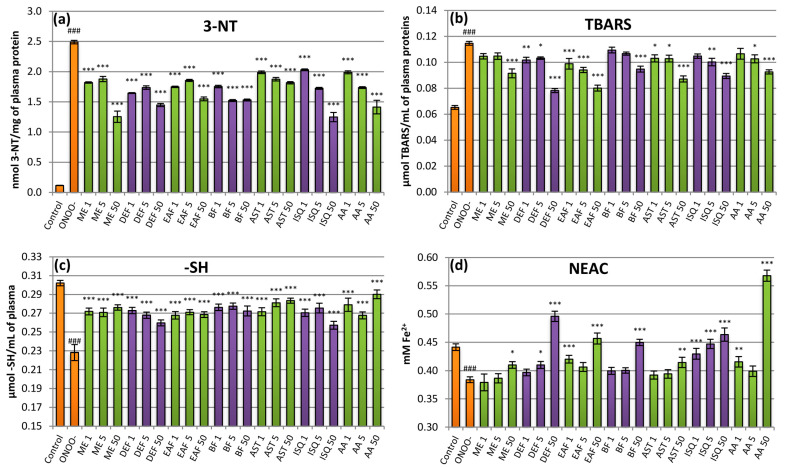
Effects of the investigated analytes on human plasma exposed to oxidative stress. (**a**) Effects on the nitration of plasma proteins assessed by the levels of 3-nirotyrosine (3-NT) and expressed as the 3-nitrotyrosine-containg equivalents (nmol of 3-NT-Fg/mg of plasma protein); (**b**) effects on lipid peroxidation assessed by the levels of thiobarbituric acid-reactive substances (TBARS); (**c**) effects on plasma protein thiol levels (-SH); (**d**) effects on the non-enzymatic antioxidant capacity (NEAC) of plasma, assessed by ferric reducing ability of plasma (FRAP). Results presented as means ± SE (*n* = 12–14). Statistical differences: ### *p* < 0.001 for control plasma versus ONOO^−^-treated plasma (without the investigated analytes); * *p* < 0.05, ** *p* < 0.01, *** *p* < 0.001 for ONOO^−^-treated plasma in the presence of the analytes versus ONOO^−^-treated plasma without the analytes. Analytes: ME—methanol extract; DEF—diethyl ether fraction; EAF—ethyl acetate fraction; BF—*n*-butanol fraction; AST—astragalin; ISQ—isoquercitrin; AA—ascorbic acid; number after analyte symbol indicates concentration in μg/mL.

In the present paper, oxidative stress was induced by adding to the human plasma samples a bolus of ONOO^–^, obtained by chemical synthesis [37]. The addition caused measurable changes in plasma parameters, reflecting the oxidative/nitrative damage inflicted by the species. Levels of the markers of protein nitration (3-NT) and lipid peroxidation (TBARS) were significantly (*p* < 0.001) increased (Figure 4a,b), while the natural antioxidant properties of plasma were depleted. The latter was evidenced by a significant (*p* <0.001) decrease in thiol levels and NEAC (Figure 4c,d). The extracts added as a protective measure substantially reduced the observed damage. In the case of 3-NT, all the investigated extracts revealed strong protective activity in the whole range of the tested concentrations, covering both low levels of 1–5 µg/mL, possible to obtain in vivo after oral intake of polyphenols, as well as higher levels of 50 µg/mL that might be possible to achieve, e.g., after topical application [4]. The nitration inhibition reached from about 27–33% at 1 µg/mL to 38–50% at 50 µg/mL, depending on the extract, and was similar to that obtained in the presence of AA (Figure 4a). The impact on the TBARS levels was also significant. However, the extracts less active in scavenging assays (ME, BF) were effective only at 50 µg/mL, while the fractions with the highest anti-ROS potential (DEF, EAF) significantly reduced lipid oxidation, even at the lowest levels. Compared to the ONOO^–^-treated plasma, the extracts diminished the ONOO^–^-triggered lipid peroxidation by up to 70%. Those results were superior to the values obtained by AA that reduced the ONOO^–^-inflicted rise in TBARS levels by up to 45% at 50 µg/mL (Figure 4b). In the thiol protection test, all extracts were effective in all concentrations used, and the thiol depletion in their presence was reduced by 42–67%. In that assay, AA prevented oxidative damage by 53–83% (Figure 4c). In the NEAC assay, the protective effects of the analytes were the least marked. At 1–5 µg/mL, only the polyphenol-richest fractions EAF and DEF were active. However, at 50 µg/mL, the effects were noticeable for all extracts, and in the case of DEF and EAF, the NEAC was even elevated above its initial level in control plasma. Interestingly, a similar activity profile, with potent effects only at 50 µg/mL, was also revealed for AA (Figure 4d). A possible explanation of the smaller response to the analytes in that assay might be the relatively weak reactivity of ferrous ions used to measure the antioxidant status.

The difference in the activity between kaempferol and quercetin derivatives noticed in the scavenging tests might also, to some extent, be observed in the plasma model. It was especially evident in the protein nitration and NEAC assays, where isoquercitrin exhibited significantly better protective effects than astragalin (Figure 4a,d). On the other hand, the activity of both compounds was similar in TBARS and thiol oxidation tests (Figure 4b,c). Such results may be caused by additional interactions between the investigated flavonoids and plasma components or by the influence of mechanisms other than direct ROS scavenging.

Nevertheless, the results show that both the extracts and their constituents may indeed prevent oxidative/nitrative damage caused by the excess of ROS. At least part of that activity may be connected with the direct scavenging properties, although other mechanisms might also be involved. As some results suggest, the injury of vessel walls precedes the development of further disorders such as valves insufficiency and vein dilatation [10]. The association between the oxidative stress and incidence of varicose vein complications such as venous ulcers and thrombophlebitis has also been observed [10,11]. Moreover, the levels of 3-NT, in particular, in some of the studies were strongly correlated with the severity of the venous disorders [36]. Thus, the protective activity of the extracts of horse chestnut flower might be one of the mechanisms of its efficacy, both in the development and progress of venous insufficiency.

### 2.5. Influence on Plasma Haemostasis Parameters

Increased risk of thrombotic events in patients with varicose veins often justifies the use of anti-coagulant or anti-aggregatory drugs [38]. Plant extracts used in adjuvant therapy might also have similar properties. Such properties may benefit the treatment; however, they might also cause unwanted and dangerous interactions [39]. For that reason, in the last part of the study, we investigated basic anti-platelet and anticoagulant effects of the extracts.

**Figure 5:**
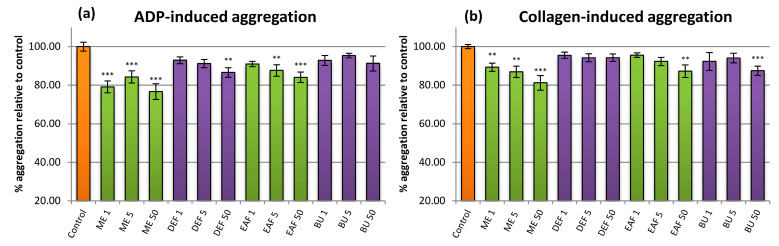
Evaluation of the anti-platelet effects of the examined analytes. The hemostatic response of blood platelets (aggregation in the platelet-rich plasma, PRP) was induced by (**a**) ADP and (**b**) collagen. Results presented as means ± SE (*n* = 11). Statistical differences: ** *p*< 0.01, *** *p*< 0.001 for control platelets versus platelets incubated with analytes. Analytes: ME—methanol extract; DEF—diethyl ether fraction; EAF—ethyl acetate fraction; BF—*n*-butanol fraction; number after analyte symbol indicate concentration in μg/mL.

The anti-platelet effects were inspected in platelet-rich plasma, in which the aggregation was induced by ADP or collagen (Figure 5). In both cases, the highest inhibition rate was achieved for ME, active in the whole range of concentrations tested (1–50 µg/mL), with a maximum inhibition of about 20%. Other extracts were less effective (revealing up to 15% inhibition), with significant results obtained almost exclusively at the highest concentration levels.

In the literature, there are some reports about significant anti-platelet activity of phenolics such as those present in the investigated extracts. Kaempferol, for example, was found to be a strong inhibitor of platelet aggregation induced by collagen, acting mainly by inhibiting NADPH oxidase and the subsequent decrease in O_2_^•−^ generation. [40]. In the present study, despite the high content of kaempferol derivatives, the investigated extracts had no effects on platelet aggregation or possessed only slight anti-aggregatory potential. One of the reasons might be that glycosidation of kaempferol hinders its ability to interact with the enzyme. On the other hand, since O_2_^•−^ and other ROS are known to act as second messengers in platelet activation pathways [41], the antioxidant substances might potentially interrupt the signaling cascades due to their scavenging properties. Our results show that the extracts can only mildly impair internal platelet communication at physiologically relevant concentrations. On the other hand, due to their antioxidant properties, the extracts might be still able to prevent excessive platelet activation connected with oxidative stress within the insufficient vessels, although further studies are required to confirm that hypothesis.

The coagulation cascade is a complex process, involving sequential activation of a series of proenzymes, leading finally to thrombin activation, proteolysis of blood plasma fibrinogen, its polymerization, and fibrin clot formation [42]. Thus, there are many molecular targets through which plant extracts may act. Evaluation of clotting times is a fast and straightforward screening test that captures the strongest effects, which might have the most spectacular impact on the activity of an analyte.

**Figure 6:**
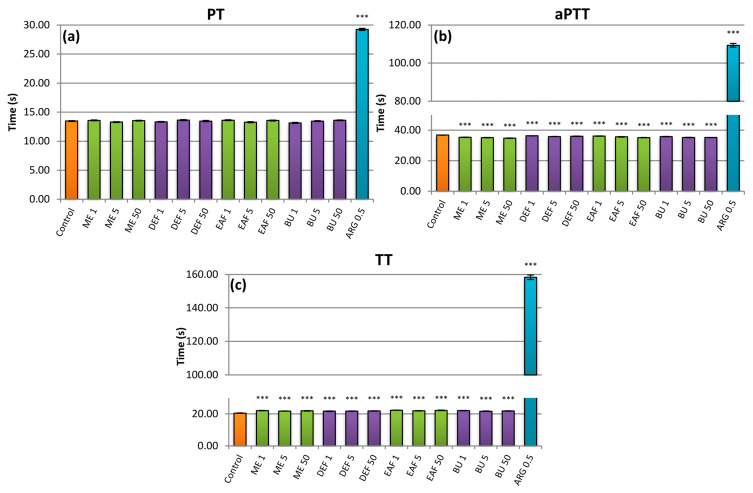
Effects of the investigated analytes on coagulation times: (**a**) prothrombin time (PT), (**b**) activated partial thromboplastin time (aPTT), (**c**) thrombin time (TT). Results presented as means ± SE (*n* = 10–12). Statistical differences: *** *p* < 0.001 for control plasma versus plasma incubated with analytes. Analytes: ME—methanol extract; DEF—diethyl ether fraction; EAF—ethyl acetate fraction; BF—*n*-butanol fraction; ARG—argatroban; number after analyte symbol indicates concentration in μg/mL.

In the present study, the effects of the studied extracts on prothrombin time (PT), activated partial thromboplastin time (aPTT), and thrombin time (TT) were measured in comparison to a standard anticoagulant, argatroban (Figure 6). The results showed no impact on PT (Figure 6a), only a small statistically significant decrease in aPTT for all extracts in all concentrations (Figure 6b), and a similarly slight increase in TT also for all analytes tested (Figure 6c). In comparison to the positive control, the results were negligible, and no dose-dependency of the effects was noticed. Thus, the capacities of the extracts seem to be of low clinical relevance, and the anticoagulant mechanism might not be considered a factor behind their efficacy. On the other hand, such results demonstrate the potential safety of the extracts’ application in combination with standard anticoagulant drugs.

## 3. Materials and Methods

### 3.1. General

The analytical grade methanol, diethyl ether, ethyl acetate, and *n*-butanol for extraction were purchased from Avantor Performance Materials (Gliwice, Poland). HPLC-grade solvents (acetonitrile, formic acid, ortho-phosphoric acid) used for UHPLC and HPLC analyses were obtained from Avantor Performance Materials and Sigma-Aldrich (Seelze, Germany/St. Louis, MO, USA). All reagents for scavenging assays were purchased from Sigma-Aldrich. ONOO^−^ was synthesized according to Pryor et al. [37]. Pierce BCA Protein Assay Kit was obtained from Thermo Scientific (Waltham, MA, USA). All immune reagents for 3-NT detection were purchased from Abcam (Cambridge, United Kingdom). Redistilled water was used in all analyses. For chemical tests requiring constant temperature, the samples were incubated in a BD 23 incubator (Binder, Tuttlingen, Germany). Scavenging assays and activity tests in blood plasma models were performed in 96-well microplates using a SPECTROStar Nano microplate reader (BMG LabTech, Ortenberg, Germany).

### 3.2. Plant Material

Flower of *Aesculus hippocastanum* L. were collected from plants growing in their natural habitat in Lodz in June 2017. The plant material was authenticated by prof. M. Olszewska. A voucher sample was deposited in the herbarium of the Department of Pharmacognosy, Medical University in Lodz, Poland (KFG/HB/AHIP_1701_F). The collected plant material was dried and stored under ambient conditions. Prior to the analyses, a representative sample was powdered using electric grinder and sieved (0.315 mm).

### 3.3. Preparation of the Extracts

A sample of plant material (250 g) was first defatted by extraction with chloroform (1 L) in Soxhlet apparatus (30 h). The pellet was then refluxed with methanol (5 × 750 mL × 3 h) to yield (after evaporation of the solvent) ME (53 g dw). A portion of ME (3 g) was set aside for further analyses, and the rest was redissolved in water and subjected to fractionated extraction with diethyl ether, ethyl acetate, and *n*-butanol, subsequently. The solvents were evaporated in vacuum and then lyophilized using an Alpha 1–2/LD Plus freeze dryer (Christ, Osterode am Harz, Germany) to remove residual water. In the process, the following dry fractions were obtained: DEF, EAF, and BF.

### 3.4. Qualitative UHPLC–PDA–ESI–TQ–MS/MS Profiling

The UHPLC–PDA–ESI–TQ–MS/MS analysis was performed on an LCMS-8050 system (Shimadzu, Kyoto, Japan) consisting of Nexera-X2 chromatograph (two high-pressure gradient pumps, an autosampler, a column compartment, a diode array detector) and MS triple quadrupole detector. Separations were carried out on a Luna Omega C18 column (1.6 µm, 150 × 2.1 mm; Phenomenex, Torrance, CA, USA) at 35 °C. The mobile phase consisted of solvent A (0.1% *v*/*v* formic acid) and solvent B (acetonitrile) with the elution profile as follows: 0–0.5 min, 5% B in A (*v/v*); 0.5–25.0 min, 5–30% B; 25.0–25.1 min, 30–95% B; 25.1–30.0 min, 95% B; 30.0–30.1 min, 95–5% B; 30.1–35.0 min, 5% B (equilibration). The flow rate was 0.25 mL/min. Samples of the extracts were dissolved in methanol/water 7:3 (*v*/*v*); the obtained solutions were filtered through a PTFE syringe filter (13 mm, 0.2 µm, Vitrum) and injected into the UHPLC system (1 µL). UV–VIS spectra were recorded over the range of 220–400 nm. The MS data were collected in Product Ion Scan (negative ions) mode from *m/z* 150 to 2000 using ionization energies of 15, 25, 35, and 45 eV. The peaks were identified by comparison of their retention times, UV spectra, and MS spectra with the reference substances or literature data.

### 3.5. Quantitative HPLC–PDA Analysis

The HPLC–PDA analysis was performed using a Prominence-I HPLC system (Shimadzu) equipped in a quaternary pump, an autosampler, a column compartment, and a diode array detector. Separations were carried out using Ascentis Express C18 column (3 µm, 150 × 4.6 mm; Supelco, Bellefonte, PA, USA) at 35 °C. The mobile phase consisted of solvent A (0.5% orthophosporic acid *w/v*), solvent B (acetonitrile), and solvent C (tetrahydrofuran), with the elution profile as presented in Table 4. The flow rate was 1 mL/min. UV–VIS spectra were recorded over the range of 220–400 nm. The applied method was validated in terms of linearity, precision, accuracy, and sensitivity (for details, see the Supplementary Materials). Accurately weighted samples of the extracts were dissolved in methanol/water 7:3 (*v*/*v*, 10 mL); the obtained solutions were filtered through a PTFE syringe filter (13 mm, 0.2 µm, Vitrum) and injected into the HPLC system (5 µL). The contents of the investigated phenolics were determined using calibration curves of appropriate reference substances purchased or isolated in our department (Table S1). For details concerning the method development and validation, see the Supplementary Materials.

### 3.6. Antioxidant Activity in Chemical Models

The scavenging capacity of the analytes towards O_2_^•−^, H_2_O_2_, OH^•^, and ONOO^–^ were assessed using spectrophotometric microplate procedures, as described previously [4]. AA was used as positive control. For all assays, SC_50_ values (the concentration of the analyte that decreases the initial amount of the oxidant by 50%) were obtained from concentration-scavenging curves (5–10 calibration points) and expressed in µg/mL with respect to the dry weight of the extract or standard as well as in AAE (mmol AA/per g of dry weight of the extract or standard).

### 3.7. Preparation of Plasma Samples

Blood plasma was obtained from buffy coat units purchased from the Regional Centre of Blood Donation and Blood Treatment in Lodz, Poland. Fresh whole blood for experiments on blood platelets was obtained from healthy volunteers and collected at the Ludwik Rydygier Medical Center in Lodz, Poland. Informed consent was obtained from all subjects involved in the study. Experiments were conducted in accordance with the Declaration of Helsinki. The study design was verified and approved by the committee on the Ethics of Research at the University of Lodz (12/KBBN-UŁ/I/2017). Blood was collected onto CPD (citric acid (3.27 g/L), sodium citrate (26.3 g/L), monobasic sodium phosphate (2.11 g/L), and dextrose (25.5 g/L)) and centrifuged to obtain plasma (3000× *g*, 15 min) or platelet-rich plasma (PRP; 250× *g*, 10 min).

### 3.8. Antioxidant Activity in Human Plasma Model

Plasma samples were pre-incubated with the examined analytes at final concentrations of 1–50 µg/mL for 5 min at 37 °C, and then treated with 150 µM (the FRAP assay) or 100 µM (the remaining experiments on blood plasma) of ONOO^–^. Control samples were prepared with plasma without the analytes or ONOO^–^. No pro-oxidative effect was found in the experiments with plasma and the analytes only (without ONOO^–^). Non-enzymatic antioxidant capacity (NEAC) of plasma was evaluated by measuring its ferric-reducing ability (FRAP) according to Marchelak et al. [24], and the results were expressed in millimolars of Fe^2+^ equivalents. The 3-NT-containing proteins were detected by the competitive ELISA (enzyme-linked immunosorbent assay), according to Bijak et al. [43]. The levels of nitrated proteins were expressed in the equivalents of 3-nitrotyrosine-containing fibrinogen (3-NT-Fg) (in nmol/mg of plasma proteins). The concentration of free -SH groups in plasma was measured spectrophotometrically using Ellman’s reagent, according to Bijak et al. [25] and expressed in µmol/mL of plasma. The TBARS levels were determined according to Kolodziejczyk et al. [44] and expressed in µmol TBARS/mL of plasma. In all experiments, AA was used as a positive control.

### 3.9. Influence on Plasma Hemostasis Parameters

Platelet aggregation was measured in PRP, using the Chrono-Log 490 aggregometer (CHRONO-LOG, Havertown, PA, USA). PRP samples were pre-incubated with the examined extract/standards at the final concentrations of 1–50 µg/mL for 15 min at 37 °C and transferred into aggregometer cuvettes. Aggregation was induced by ADP (at the final concentration of 10 µM) or collagen (at the final concentration of 2 µg/mL). Control samples were untreated with the examined analytes.

TT, PT, and aPTT were measured in fresh blood plasma with the use of Kselmed K-3002 Optic coagulometer (Grudziądz, Poland), using reagents purchased from Diagon Kft. (Budapest, Hungary). Plasma samples were pre-incubated with the examined analytes at the final concentration of 1–50 µg/mL for 15 min at 37 °C. Control samples were untreated with the examined analytes. Argatroban was used as a positive control.

### 3.10. Statistical analysis

The quantitative results were expressed as means (*n* ≥ 3) ± standard error (SE). Normality of the distribution of the results was verified using the Shapiro–Wilk test, and the homogeneity of variances using the Levene’s test. The significance of differences between samples and controls was determined with one-way ANOVA followed by the post hoc Tukey’s test for multiple comparisons. All calculations were performed using the Statistica12Pl software for Windows (StatSoft Inc., Krakow, Poland), with *p*-values less than 0.05 being regarded as significant.

## 4. Conclusions

The present work allowed for the most thorough characterization of the polyphenolic profile of horse chestnut flower published thus far. Most of the phenolic acids and flavanols were found in the plant material for the first time, while the information concerning flavonol derivatives, the main active flower components, was significantly broadened. Moreover, the fractionated extraction was for the first time applied to selectively extract the active polyphenols from the plant material and obtain the polyphenol-enriched fractions with increased biological capacity. Diethyl ether and ethyl acetate were found to be the most effective extractants for that procedure, as they not only improved the content of phenolics by three to four times, but also changed the phytochemical profile promoting the constituents with potentially higher bioavailability. The bioactivity study indicated that the extracts and their constituents may protect plasma biomolecules, such as lipids and proteins, from oxidative/nitrative damage, and that at least part of that activity might be connected with direct ROS scavenging. Such activity might be relevant for alleviating the symptoms of varicose veins and similar conditions, being one of the mechanisms behind the efficacy of horse chestnut flower in those conditions. On the other hand, the anti-coagulant and anti-platelet capacity does not seem of clinical relevance, and the extracts should be safe to use in combination with drugs impacting the coagulation process.

## Figures and Tables

**Table 1:** UHPLC–PDA–ESI–MS/MS data of compounds detected in dry extracts from the flower of *A. hippocastanum*.

No	Analyte	R_t_ (min)	UV λ_max_ (nm)	[M−H]^–^	Fragmentary Ions
1	gallic acid ^a,b,c^	2.43	270	169	131
2	protocatechuic acid ^a,b,c^	4.87	258, 293	153	109
3	chlorogenic acid ^a,b,c^	5.85	324	353	191, 179
4	coumaric acid derivative I ^b,c^	7.32	311	487	427, 163, 145, 119
5	*p*-hydroxybenzoic acid ^a,b,c^	7.65	252	137	
6	coumaroylquinic acid ^b,c^	7.87	329	337	163, 191
7	coumaric acid derivative II ^b,c^	8.76	268, 314	487	427, 163, 145, 119
8	cryptochlorogenic acid ^a,b,c^	9.24	322	353	179, 191
9	coumaric acid derivative III ^b,c^	9.58	305	487	427
10	caffeic acid ^a,b,c^	9.72	319	179	135
11	Fraxin ^a,b^	10.03	304	369	
12	procyanidin B2 ^a,b^	10.37	277	577	425, 289
13	coumaric acid derivative IV ^b,c^	10.46	314	487	265, 163, 145, 119
14	unidentified	10.85		331	151
15	(−)-epicatechin ^a,b^	11.17	278	289	245, 205
16	procyanidin trimer ^b^	12.48	278	863	447, 289
17	*p*-coumaric acid ^a,b,c^	13.18	308	163	119
18	quercetin dihexoside ^b^	13.55	263, 351	625	301, 445
19	coumaric acid isomer ^b^	13.94	298	163	
20	quercetin hexoside pentoside ^b^	14.70	266, 353	595	301
21	procyanidin dimer A ^b^	15.03	276	575	449, 289
22	kaempferol dihexoside	15.16	264, 349	609	429, 285
23	procyanidin dimer A ^b^	15.26	275	575 (245)	
24	quercetin 3-*O*-sophoroside ^b,c^	15.64	264, 347	609	300
25	quercetin 3-*O*-rutinoside (rutin) ^a^	15.92	262, 355	609	301
26	quercetin 3-*O*-β-d-galactopyranoside (hiperoside) ^a,b^	16.23	260, 354	463	301
27	quercetin 3-*O*-β-d-glucopyranoside (isoquercetin) ^a^	16.57	253, 353	463	301
28	kaempferol hexoside pentoside ^b^	16.66	260, 351	579	285
29	procyanidin A2 ^a,b^	16.79	277	575	423, 289
30	kaempferol hexoside rhamnoside ^b,c^	17.01	263, 351	593	285
31	kaempferol 3-*O*-β-d-galactopyranoside (trifolin) ^a,b,c^	17.81	263, 348	447	285
32	kaempferol 3-*O*-rutinoside ^a^	17.89	264, 347	593	285
33	quercetin 3-*O*-α-l-arabinofuranoside (avicularin) ^a^	18.26	257, 353	433	301
34	kaempferol 3-*O*-β-d-glucopyranoside (astragalin) ^a^	18.6	264, 347	447	285
35	quercetin 3-*O*-α-l-rhamnopyranoside (quercitrin) ^a,b^	18.79	260, 349	447	301
36	kaempferol pentoside ^b,c^	19.43	264, 324	417	285
37	kaempferol acetylhexoside ^b,c^	20.23	264, 347	489	285
38	kaempferol 3-*O*-α-l-arabinofuranoside (juglanin) ^a^	20.52	264, 347	417	285
39	kaempferol 3-*O*-α-l-rhamnopyranoside (afzelin) ^a^	21.25	263, 342	431	285
40	tricaffeoyl spermidine ^b,c^	22.57	322	630	468, 306
41	quercetin ^a^	24.44	258, 370	301	151, 273
42	dicaffeoyl-feruloyl spermidine ^b,c^	24.75	321	644	508, 372
43	kaempferol ^a^	26.91	262, 366	285	131

^a^ Confirmed by comparison with authentic standard; ^b^ detected for the first time in *A. hippocastanum* flower; ^c^ detected for the first time in *A. hippocastanum*; R_t_, retention time; UV λ_max_, absorbance maxima in PDA spectrum; [M−H]^−^, *m/z* of deprotonated molecule in MS spectra recorded in a negative mode.

**Table 2:** Content of the major polyphenols in the dry extracts from the flower of *A. hippocastanum*.

No ^a^	Analyte	Content (mg/g)			
		ME	DEF	EAF	BF
2	protocatechuic acid ^b^	0.50 ± 0.07 ^A^	15.00 ± 0.40 ^B^	0.97 ± 0.11 ^A^	n.d.
3	chlorogenic acid ^b^	0.52 ± 0.01 ^A^	n.d.	n.d.	2.68 ± 0.04 ^B^
4	coumaric acid derivative I ^c^	n.d.	n.d.	n.d.	0.56 ± 0.01
*5*	*p*-hydroxybenzoic acid ^b^	n.d.	5.85 ± 0.13	n.d.	n.d.
7	coumaric acid derivative II ^c^	n.d.	n.d.	n.d.	0.56 ± 0.01
13	coumaric acid derivative IV ^c^	n.d.	n.d.	n.d.	0.61 ± 0.02
15	(−)-epicatechin ^b^	0.87 ± 0.03 ^A^	4.45 ± 0.13 ^B^	15.69 ± 0.89 ^C^	n.d.
16	procyanidin trimer ^d^	2.11 ± 0.06 ^A^	n.d.	16.66 ± 0.05 ^B^	n.d.
17	p-coumaric acid ^b^	n.d.	8.81 ± 0.14	n.d.	n.d.
18	quercetin 3-*O*-sophoroside ^e^	n.d.	n.d.	n.d.	2.53 ± 0.05
19	coumaric acid isomer ^c^	n.d.	0.91 ± 0.1	n.d.	n.d.
20	quercetin hexoside pentoside ^e^	n.d.	n.d.	n.d.	3.74 ± 0.13
22	kaempferol dihexoside	n.d.	n.d.	n.d.	6.60 ± 0.09
23	procyanidin dimer A ^d^	1.88 ± 0.03 ^A^	n.d.	4.52 ± 0.26 ^B^	n.d.
25	quercetin 3-*O*-rutinoside (rutin) ^b^	1.64 ± 0.05 ^A^	n.d.	2.55 ± 0.02 ^B^	13.21 ± 0.15 ^C^
27	quercetin 3-*O*-β-d-glucopyranoside (isoquercetin) ^b^	3.89 ± 0.04 ^B^	4.63 ± 0.07 ^C^	27.66 ± 0.15 ^D^	2.26 ± 0.04 ^A^
28	kaempferol hexoside pentoside ^f^	2.32 ± 0.04 ^A^	n.d.	n.d.	15.62 ± 0.09 ^A^
29	procyanidin A2 ^b^	2.35 ± 0.05 ^A^	12.25 ± 0.26 ^B^	20.77 ± 0.68 ^C^	n.d.
30	kaempferol hexoside rhamnoside ^f^	n.d.	n.d.	2.08 ± 0.03 ^A^	6.27 ± 0.12 ^B^
31	kaempferol 3-*O*-β-d-galactopyranoside (trifolin) ^g^	1.52 ± 0.02 ^A^	2.95 ± 0.11 ^B^	n.d.	n.d.
32	kaempferol 3-*O*-rutinoside ^b^	16.31 ± 0.12 ^B^	2.00 ± 0.01 ^A^	54.77 ± 0.78 *^C^*	81.97 ± 0.59 ^D^
33	quercetin 3-*O*-α-l-arabinofuranoside (avicularin) ^b^	0.95 ± 0.02 ^A^	10.32 ± 0.23 ^C^	3.61 ± 0.15 ^B^	n.d.
34	kaempferol 3-*O*-β-d-glucopyranoside (astragalin) ^b^	29.38 ± 0.22 ^B^	81.98 ± 1.93 ^C^	208.23 ± 1.95 ^D^	4.92 ± 0.05 ^A^
35	quercetin 3-*O*-α-l-rhamnopyranoside (quercitrin) ^b^	0.83 ± 0.01 ^A^	6.49 ± 0.15 ^C^	5.89 ± 0.11 ^B^	n.d.
36	kaempferol pentoside ^h^	n.d.	2.85 ± 0.05 ^B^	1.41 ± 0.01 ^A^	n.d.
37	kaempferol acetylhexoside ^g^	2.37 ± 0.03 ^B^	1.82 ± 0.07 ^A^	16.64 ± 0.20 ^D^	4.92 ± 0.05 ^C^
38	kaempferol 3-*O*-α-l-arabinofuranoside (juglanin) ^b^	2.96 ± 0.03 ^A^	53.89 ± 1.00 ^C^	8.01 ± 0.03 ^B^	n.d.
39	kaempferol 3-*O*-α-l-rhamnopyranoside (afzelin) ^b^	3.00 ± 0.02 ^A^	52.33 ± 1.07 ^C^	15.59 ± 0.28 ^B^	n.d.
40	tricaffeoyl spermidine ^i^	0.22 ± 0.01	n.d.	1.24 ± 0.05	n.d.
41	quercetin ^b^	n.d.	8.01 ± 0.17	n.d.	n.d.
42	dicaffeoyl-feruloyl spermidine ^i^	0.23 ± 0.01	n.d.	1.04 ± 0.07	n.d.
43	kaempferol ^b^	0.39 ± 0.01 ^A^	32.93 ± 0.58 ^C^	4.70 ± 0.17 ^B^	n.d.
	Total phenolic acid derivatives	1.46 ± 0.05	30.57 ± 0.54	3.25 ± 0.01	4.41 ± 0.06
	Total flavanols	7.22 ± 0.11	16.70 ± 0.35	57.64 ± 1.19	n.d.
	Total flavonoids	65.58 ± 0.44	260.20 ± 4.80	353.16 ± 1.06	142.04 ± 1.25
	Total phenolics	74.26 ± 0.57	307.46 ± 5.55	414.06 ± 1.27	146.45 ± 1.30

^a^ Compound numbering according to Table 1. Compounds not included in the quantification were present in all extracts at concentrations below the limits of quantification or determination; ^b^ quantified using calibration curve of the authentic standard; ^c^ quantified using calibration curve of coumaric acid; ^d^ quantified using calibration curve of procyanidin A2; ^e^ quantified using calibration curve of rutin; ^f^ quantified using calibration curve of kaempferol 3-rhamnoside; ^g^ quantified using calibration curve of astragalin; ^h^ quantified using calibration curve of juglanin; ^i^ quantified using calibration curve of caffeic acid. Data are presented as means ± SE (*n* = 3); ANOVA and post hoc tests for multiple comparisons were run for each constituent separately, and the values statistically different at α = 0.05 were labeled with different capital (A–D); n.d.—not determined, below quantification or detection limit; ME—methanol extract; DEF—diethyl ether fraction; EAF—ethyl acetate fraction; BF—*n*-butanol fraction.

**Table 3:** Scavenging activity of the dry extracts from the flower of *A. hippocastanum* towards selected reactive oxygen species.

Analyte	SC_50_ [µg/mL]			
	H_2_O_2_	OH^•^	O_2_^−•^	ONOO^−^
MED	157.78 ± 5.79 ^G^	532.57 ± 13.37 ^G^	59.09 ± 1.55 ^E^	>400
DEF	44.20 ± 1.98 ^D^	134.93 ± 3.79 ^D^	24.18 ± 2.07 ^C^	173.42 ± 5.19
EAF	34.64 ± 0.55 ^C^	117.33 ± 2.41 ^C^	15.22 ± 0.94 ^B^	154.02 ± 5.64
BF	70.44 ± 2.42 ^E^	189.11 ± 7.85 ^F^	30.04 ± 3.07 ^D^	286.19 ± 6.68
Astragalin	91.86 ± 3.01 ^F^	102.46 ± 8.09 ^B,C^	>500	>400
Isoquercitrin	14.28 ± 0.71 ^B^	62.22 ± 2.72 ^A^	19.21 ± 0.83 ^B^	69.48 ± 1.88 ^A^
(−)-Epicatechin	8.41 ± 0.30 ^A^	99.53 ± 0.85 ^B^	7.89 ± 0.99	
Ascorbic acid	11.59 ± 0.81 ^B^	151.33 ± 2.98 ^E^	5.76 ± 0.96 ^A^	67.91 ± 2.58 ^A^

Data presented as means ± SE (*n* = 5). ANOVA and post hoc tests for multiple comparisons were run foreach ROS separately, and the values statistically different at α = 0.05 were labeled with different capital (A–G); ME—methanol extract; DEF—diethyl ether fraction; EAF—ethyl acetate fraction; BF—*n*-butanol fraction.

**Table 4:** The optimized elution profile for quantitative analysis of polyphenols in the flowers of *A. hippocastanum*.

Time (min)	Solvent A (%)	Solvent B (%)	Solvent C (%)
0.0–1.0	92	1	7
1.0–20.0	92–68	1–25	7
20.0–25.0	68–18	25–75	7
25.0–30.0	18	75	7
30.1–35.0	92	1	7

Solvent A: 0.5% aqueous solution of orthophosphoric acid (*w/v*); solvent B: acetonitrile; solvent C: tetrahydrofuran.

## Data Availability

Data is contained within the article and supplementary material.

## Supplementary Materials

The following are available online at https://www.mdpi.com/article/10.3390/ph14121301/s1, Figure S1: title. Table S1: Reference substances used for quantification of polyphenols in the extracts from flowers of *A. hippocastanum*. Table S2: Linearity and sensitivity data for the proposed HPLC–PDA method. Table S3: Precision and accuracy data for the proposed HPLC–PDA method.
