# Supplementary material for: Bioactivity Potential of Aesculus hippocastanum L. Flower: Phytochemical Profile, Antiradical Capacity and Protective Effects on Human Plasma Components under Oxidative/Nitrative Stress In Vitro
Source: Pharmaceuticals (Basel). 2021 Dec 14;14(12):1301. doi: 10.3390/ph14121301 (PMC8706066; doi:10.3390/ph14121301)
Supplement: Supplementary file 1 [file pharmaceuticals-14-01301-s001.zip › pharmaceuticals-1505822-supplementary.pdf]

pharmaceuticals

Article

Horse Chestnut Flower Extracts with Strong Antiradical Potential Protect Human Plasma Components against Oxidative/Nitrative Damage in vitro

Aleksandra Owczarek [1,*], Joanna Kołodziejczyk-Czepas [2], Paulina Marczuk [2], Julia Siwek [1], Katarzyna Wąsowicz [1], Monika Anna Olszewska [1]

1 Department of Pharmacognosy, Faculty of Pharmacy, Medical University of Lodz, 90-151 Lodz, Poland; monika.olszewska@umed.lodz.pl (M.A.O.), julia.siwek@stud.umed.lodz.pl (J.S.), katarzyna.wasowicz6@gmail.com (K.W.)
2 Department of General Biochemistry, Faculty of Biology and Environmental Protection, University of Lodz, 90-236 Lodz, Poland; joanna.kolodziejczyk@biol.uni.lodz.pl (J.K.-C.); mmarczuk.paulina@gmail.com (P. M.)
* Correspondence: aleksandra.owczarek@umed.lodz.pl;

Supplementary Materials

1. Development and Validation of the HPLC-PDA Method

1.1. Method development

An HPLC-PDA method was developed for quantitative determination of the main phenolics in the investigated extracts from the flower of *A. hippocastanum*. The method was optimized on HPLC system, which is less prone to clogging than UHPLC equipment and thus has good compatibility with plant extracts [1]. To improve the efficiency of the separation a fused-core column was used allowing for separation quality comparable to that of UHPLC at only half the operating pressure [2]. Such approach, has previously been proved effective in separation of complicated phenolic matrices, e.g. in the flower of Prunus spinosa [3].

The separation process was optimized to obtain satisfactory resolution between the peaks, prioritizing the dominant constituents of the plant. Several factors were taken into account including the elution solvents (binary and ternary elution systems were tested), initial concentration of acetonitrile, gradient slope, temperature of the analysis, flow rate, and in case of ternary systems also concentration of tetrahydrofuran. The finally selected elution profile allowed for separation of the analyzed samples under 25 minutes. The method allowed for chromatographic discrimination and quantification of 33 extracts constituents, whose levels were quantifiable in at least one of the extract. Among them were 17 constituents quantified directly, using appropriate reference standard. Due to only tentative identification, the rest of the constituents could be quantified only indirectly using calibration curves of the standard with the most similar structure and molecular mass (as is marked in Table 2).

1.2. Validation of the HPLC-PDA Method

The analytical method was validated by determining the selectivity, linearity, precision, and accuracy according to the guidance of the International Council for Harmonisation [4].

The validation was performed for seventeen fully identified bark components, that had the authentic standard available, obtained either from commercial source or from previous isolation studies in the Department of Pharmacognosy (Table S1).

The selectivity of the method, as well as the peak purity, were analyzed by comparison of the retention times and UV-vis spectra with reference compounds using an automatech match system. Comparisons of the spectra's upslopes, apexes, and downslopes, as well as the peak spectral data at different wavelengths, confirmed that all analyte peaks of the real samples eluted as pure bands.

The linearity of the method for the six standards was confirmed in the whole range of concentrations used with $r > 0.999$ (Table S2). The statistical significance of the obtained regression equations was confirmed in the F-test ($p < 0.05$). Good sensitivity of the method was demonstrated by low LODs and LOQs values (Table S2).

In the precision test, the RSD values (Table S3) measured for peak area of each analyte for repeatability and intermediate precision did not exceed the predicted critical values (PRSD, 3.21-8.84%), calculated according to the requirements of AOAC International [5] using Horwitz equation, which indicated that the developed method is adequately precise. In accuracy studies, the recoveries were between 98.3% and 101.8% (Table S3) and within the limits of acceptance (92-105%) [6].

Table S1. Reference substances used for quantification of polyphenols in the extracts from the flowers of *A. hippocastanum*

Reference substance	Purity and source
protocatechuic acid	>97% (A)
chlorogenic acid	>98% (A)
(−)-epicatechin	>97% (A)
p-hydroxybenzoic acid	>99% (A)
caffeic acid	>98% (A)
p-coumaric acid	>98% (A)
procyanidin A2	>98% (A)
quercetin 3-*O*-(6"-*O*-α-L-rhamnopyranosyl)-β-D-glucopyranoside (rutin)	>98% (A)
quercetin 3-*O*-β-D-glucopyranoside (isoquercitrin)	>97% (A)
kaempferol 3-*O*-(6"-*O*-α-L-rhamnopyranosyl)-β-D-glucopyranoside	>98% (B)
kaempferol 3-*O*-β-D-glucopyranoside (astragalin)	>98% (B)
quercetin 3-*O*-α-L-arabinofuranoside (avicularin)	>98% (B)
quercetin 3-*O*-α-L-rhamnopyranoside (quercitrin)	>98% (B)
kaempferol 3-*O*-α-L-arabinofuranoside (juglanin)	>98% (B)
kaempferol 3-*O*-α-L-rhamnopyranoside (afzelin)	>98% (B)
quercetin	>95% (A)
kaempferol	>96% (A)

A, compounds purchased from Sigma Aldrich (Seelze, Germany/St. Louis, MO, USA) or Phytolab (Vestenbergsgreuth, Germany); B, compounds isolated in the Department of Pharmacognosy;

Table S2. Linearity and sensitivity data for the proposed HPLC-PDA method.

**Analyte**	**t_R ± SD (min)**	**λ (nm)**	**Linearity**			**Sensitivity**	
			Linear regression	r	Linear range (μg/mL)	LOD (μg/mL)	LOQ (μg/mL)
protocatechuic acid	5.25 ± 0.04	260	$y = 15523.51x$	0.9998	2.06-130.2	0.11	0.34
chlorogenic acid	5.84 ± 0.04	325	$y = 14863.30x$	0.9999	1.25-62.6	0.13	0.41
(−)-epicatechin	6.72 ± 0.05	280	$y = 3078.345x$	0.9991	2.07-103.6	0.63	2.07
p-hydroxybenzoic acid	7.24 ± 0.03	260	$y = 26772.65x$	0.9994	2.01-100.6	0.06	0.20
caffeic acid	8.42 ± 0.02	325	$y = 27114.70x$	0.9999	0.99-49.7	0.06	0.20
p-coumaric acid	10.78 ± 0.05	310	$y = 30948.36x$	0.9993	2.08-104.0	0.05	0.17
quercetin 3-*O*-rutinoside (rutin)	10.89 ± 0.07	350	$y = 7220.492x$	0.9992	2.07-103.6	0.31	1.03
quercetin 3-*O*-β-D-glucopyranoside (isoquercetin)	12.02 ± 0.02	350	$y = 9373.538x$	0.9992	2.05-102.6	0.31	1.02
kaempferol 3-*O*-rutinoside	12.39 ± 0.02	350	$y = 5218.295x$	0.9993	2.10-105.0	0.42	1.40
kaempferol 3-*O*-β-D-glucopyranoside (astragalin)	13.33 ± 0.02	350	$y = 8399.799x$	0.9993	8.30-415.2	0.32	1.04
procyanidin A2	13.65 ± 0.05	280	$y = 3419.870x$	0.9991	1.26-63.4	0.38	1.26
quercetin 3-*O*-α-L-arabinofuranoside (avicularin)	13.98 ± 0.02	350	$y = 10633.93x$	0.9993	2.10-105.0	0.21	0.70
quercetin 3-*O*-α-L-rhamnopyranoside (quercitrin)	14.35 ± 0.01	350	$y = 8692.455x$	0.9998	2.00-100.2	0.31	1.00
kaempferol 3-*O*-α-L-arabinofuranoside (juglanin)	15.49 ± 0.02	350	$y = 9540.829x$	0.9991	2.07-103.8	0.31	1.03
kaempferol 3-*O*-α-L-rhamnopyranoside (afzelin)	16.00 ± 0.02	350	$y = 8671.277x$	0.9998	2.01-100.6	0.31	1.00
quercetin	20.98 ± 0.02	350	$y = 11822.71x$	0.9995	2.07-103.6	0.21	0.69
kaempferol	23.09 ± 0.01	350	$y = 13674.70x$	0.9998	2.08-104.0	0.16	0.52

t_R, retention time; λ, detection wavelength; y, peak area; x, concentration of standard in μg/mL; F-test, value of the statistical Fisher variance ratio for the experimental data. LOD, limit of detection. LOQ, limit of quantification.

Table S3. Precision and accuracy data for the proposed HPLC-PDA method.

Analyte	Precision (RSD, %)				Accuracy
	Intra-day variability		Inter-day variability		Mean recovery ± SD (%)
	t_R	Peak area	t_R	Peak area	
protocatechuic acid	0.28	0.41	0.74	2.84	101.1 ± 1.21
chlorogenic acid	0.29	0.83	0.64	2.58	99.6 ± 1.03
(−)-epicatechin	0.33	0.97	0.68	3.86	98.3 ± 0.91
p-hydroxybenzoic acid	0.24	0.71	0.42	2.12	101.3 ± 0.92
caffeic acid	0.25	0.74	0.47	3.98	101.9 ± 0.85
p-coumaric acid	0.22	0.67	0.45	2.38	100.2 ± 0.79
quercetin 3-*O*-rutinoside (rutin)	0.11	0.81	0.62	2.63	99.7 ± 0.98
quercetin 3-*O*-β-D-glucopyranoside (isoquercetin)	0.09	0.52	0.36	2.11	98.7 ± 0.81
kaempferol 3-O-rutinoside	0.11	0.62	0.39	2.96	98.5 ± 0.81
kaempferol 3-*O*-β-D-glucopyranoside (astragalin)	0.11	0.58	0.36	1.16	99.4 ± 1.01
procyanidin A2	0.19	0.92	0.39	3.98	98.5 ± 1.14
quercetin 3-*O*-α-L-arabinofuranoside (avicularin)	0.09	0.71	0.29	2.21	100.6 ± 0.89
quercetin 3-*O*-α-L-rhamnopyranoside (quercitrin)	0.13	0.81	0.24	2.40	98.8 ± 0.90
kaempferol 3-*O*-α-L-arabinofuranoside (juglanin)	0.13	0.72	0.20	2.40	99.0 ± 0.72
kaempferol 3-*O*-α-L-rhamnopyranoside (afzelin)	0.08	0.89	0.18	2.45	98.7 ± 0.83
quercetin	0.05	0.83	0.19	2.32	100.6 ± 0.88
kaempferol	0.02	0.74	0.13	2.22	99.3 ± 0.71

t_R, retention time

2. Materials and Methods

2.1. Validation

To test linearity, the stock solution of the standards were prepared in methanol-water (7:3, *v*/*v*) and serially diluted with the same solvent to six concentration levels (2%, 10%, 25%, 50%, 75%, and 100% of the stock concentration). Each replicate solution was injected into the HPLC system in triplicate. The statistical significance of the regression equations was evaluated using *F*- and *t*-tests at a 99% confidence level (Table 1).

The LOD and LOQ values were determined by further serial dilution of the standard solutions with methanol-water (7:3, *v*/*v*). The lowest concentrations with the signal-to-noise ratio (S/N) above 3 were accepted as LODs, while the levels with S/N above 10 were accepted as LOQs if the RSD values for peak area were not higher than 15%.

The repeatability (intra-day variability) and the intermediate precision (inter-day variability) were tested for retention times and peak areas using the standard solutions at 10% and 100% of the stock concentration and selected extract sample containing the given analyte at measurable levels. The repeatability was determined by triplicate analysis of each sample within 24 h, while the intermediate precision was evaluated on three non-consecutive days within two weeks.

The accuracy was tested by the standard addition/recovery procedure in the selected extract samples containing the given analyte at measurable levels. Three different levels of each standard, within the analytical range were investigated. The samples were prepared in triplicate by spiking the sample with the standard solution. The replicate samples were analysed in triplicate. The accuracy was calculated as the mean recovery of the analytes from the spiked versus the non-spiked extracts.

2.2. Statistical Analysis

The results were expressed as means ± standard deviation (SD) for replicate determinations. The statistical analyses (calculation of SD, linearity studies) were performed using the Statistica12Pl software for Windows (StatSoft Inc., Krakow, Poland), with *p* values less than 0.05 being regarded as significant.

References

1. Abrahim, A.; Al-Sayah, M.; Skrdla, P.; Bereznitski, Y.; Chen, Y.; Wu, N. Practical comparison of 2.7 μm fused-core silica particles and porous sub-2 μm particles for fast separations in pharmaceutical process development. *J. Pharm. Biomed. Anal.* **2010**, *51*, 131–137, doi:10.1016/j.jpba.2009.08.023.
2. Kirkland, J.J.; Schuster, S.A.; Johnson, W.L.; Boyes, B.E. Fused-core particle technology in high-performance liquid chromatography: An overview. *J. Pharm. Anal.* **2013**, *3*, 303–312, doi:10.1016/j.jpha.2013.02.005.
3. Marchelak, A.; Olszewska, M.A.; Owczarek, A. Simultaneous quantification of thirty polyphenols in blackthorn flowers and dry extracts prepared thereof: HPLC-PDA method development and validation for quality control. *J. Pharm. Biomed. Anal.* **2020**, *184*, 112121, doi:10.1016/j.dib.2020.105319.
4. ICH *Validation of Analytical Procedures: Text and Methodology*; Chicago, 2005;
5. AOAC International Appendix F: Guidelines for Standard Method Performance Requirements. *AOAC Off. Methods Anal.* **2016**.
6. AOAC International Appendix K: Guidelines for Dietary Supplements and Botanicals. *AOAC Off. Methods Anal.* **2013**.
